# 1414. Real-World Study of Healthcare Resource Use and Costs Associated with Inappropriate and Suboptimal Antibiotic Use Among Females with Uncomplicated Urinary Tract Infection in the United States

**DOI:** 10.1093/ofid/ofab466.1606

**Published:** 2021-12-04

**Authors:** Madison T Preib, Fanny S Mitrani-Gold, Xiaoxi Sun, Christopher Adams, Ashish V Joshi

**Affiliations:** 1 STATinMED Research, Ann Arbor, MI, USA, Ann Arbor, Michigan; 2 GlaxoSmithKline plc, Collegeville, PA, USA, Chicago, Illinois

## Abstract

**Background:**

Urinary tract infections (UTIs) are the most common outpatient infection requiring medical care in the US; but, despite Infectious Diseases Society of America 2011 guidelines for treating uncomplicated UTI (uUTI), variation in prescribing practices still exists. Few studies have used real-world data (RWD) to evaluate uUTI-associated healthcare resource use (HRU) and costs. We examined HRU and direct costs associated with appropriate *and* optimal (AP&OP) and inappropriate *or* suboptimal (IA/SO) antibiotic (AB) prescribing in females with uUTI using US RWD.

**Methods:**

This retrospective cohort study used RWD from IBM MarketScan (commercial/Medicare claims) to examine uUTI-related HRU and costs (inpatient, emergency room, outpatient, pharmacy) per index uUTI episode and during 1-year follow-up among females (age ≥ 12 years) diagnosed with uUTI from July 1, 2013–December 31, 2017 (index date). Patients had an oral AB prescription ± 5 days of the index date, and continuous health plan enrollment ≥ 6 months pre/1 year post-index date; those with complicated UTI were excluded. Patients were stratified by AB prescription as follows: AP&OP = guideline-compliant and correct duration; IA/SO = guideline non-compliant/incorrect duration or re-prescription/switch within 28 days.

**Results:**

The study included 557,669 patients. In the commercial population (n=517,664, mean age 37.7 years), fewer patients were prescribed AP&OP (11.8%) than IA/SO (88.2%) ABs, a trend also seen in the Medicare population (n=40,005, mean age 74.5 years). In both populations, adjusted average numbers of uUTI-related ambulatory visits and pharmacy claims were lower for the AP&OP cohort than the IA/SO cohort during index episode and 1-year followup (p < 0.0001, Table 1). In the commercial population, total adjusted uUTI-related costs were &194 (AP&OP) versus &274 (IA/SO; p < 0.0001); in the Medicare population, total adjusted uUTI-related costs were &253 (AP&OP) versus &355 (IA/SO; p < 0.0001) (Table 2).

Table 1. uUTI-related HRU for commercial and Medicare populations calculated using the GLM model

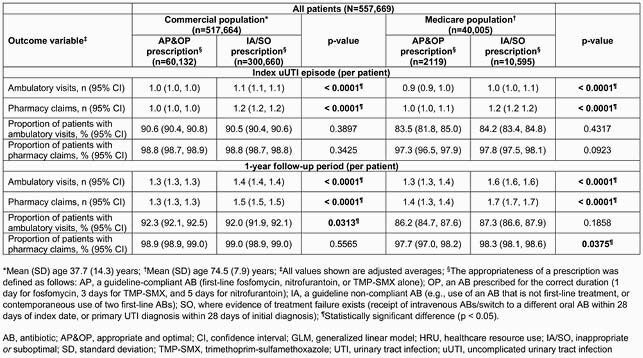

Table 2. uUTI-related costs for commercial and Medicare populations calculated using the GLM model

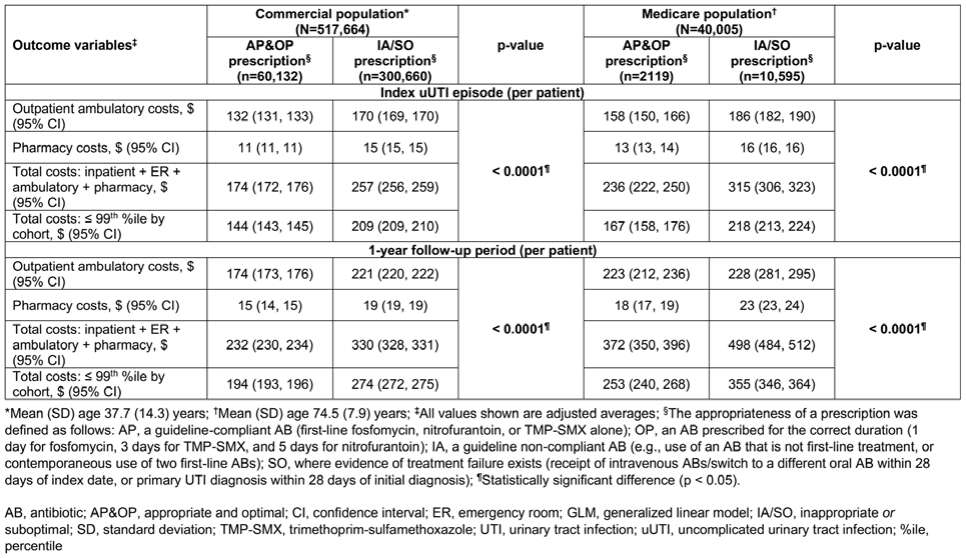

**Conclusion:**

Overall uUTI-related HRU and costs in the US were low during index episodes and follow-up. However, females with uUTI prescribed IA/SO ABs were more likely to incur higher HRU and costs than those prescribed AP&OP ABs, suggesting an unmet need for training to optimize uUTI prescribing per US guidelines.

**Disclosures:**

**Madison T. Preib, MPH**, **STATinMED Research** (Employee, Former employee of STATinMED Research, which received funding from GlaxoSmithKline plc. to conduct this study) **Fanny S. Mitrani-Gold, MPH**, **GlaxoSmithKline plc.** (Employee, Shareholder) **Xiaoxi Sun, MA**, **STATinMED Research** (Employee, Employee of STATinMED Research, which received funding from GlaxoSmithKline plc. to conduct this study) **Christopher Adams, MPH**, **STATinMED Research** (Employee, Employee of STATinMED Research, which received funding from GlaxoSmithKline plc. to conduct this study) **Ashish V. Joshi, PhD**, **GlaxoSmithKline plc.** (Employee, Shareholder)

